# A Toll-Spätzle Pathway in the Immune Response of *Bombyx mori*

**DOI:** 10.3390/insects11090586

**Published:** 2020-09-01

**Authors:** Bin Yu, Qi Sang, Guoqing Pan, Chunfeng Li, Zeyang Zhou

**Affiliations:** 1 State Key Laboratory of Silkworm Genome Biology, Southwest University, Chongqing 400715, China; yubin5868@outlook.com (B.Y.); sangqi0508@126.com (Q.S.); gqpan@swu.edu.cn (G.P.); zyzhou@swu.edu.cn (Z.Z.); 2 Chongqing Key Laboratory of Microsporidia Infection and Control, Southwest University, Chongqing 400715, China; 3 College of Life Sciences, Chongqing Normal University, Chongqing 401331, China

**Keywords:** *Bombyx mori*, innate immunity, Toll, spätzle, antimicrobial peptide

## Abstract

**Simple Summary:**

The Toll-Spätzle pathway is a crucial defense mechanism in insect innate immunity through inducing the expression of antimicrobial peptides (AMPs) against pathogens. As an important economical insect, *Bombyx mori* (*B. mori*) has been used as an insect model of Lepidoptera. However, the recognition of the Toll-Spätzle pathway in silkworm is very limited. In this study, we reported that *B. mori* Spätzle2 (BmSpz2) could be activated by pathogens. The activated BmSpz2 could bind with *B. mori* Toll11 (BmToll11) or *B. mori* Toll9-1 (BmToll9–1). Further investigations showed that overexpression of BmToll11 or BmToll9–1 could significantly upregulate AMPs expression. The findings of this study confirmed that a Toll-Spz pathway regulated immune response to microbial infection in *B. mori* and may help improve the understanding of the signaling pathways involved in the innate immune responses of Lepidoptera insect.

**Abstract:**

The Toll-Spätzle pathway is a crucial defense mechanism in insect innate immunity, it plays an important role in fighting against pathogens through the regulation of antimicrobial peptide gene expression. Although *Toll* and *Spätzle* (*Spz*) genes have been identified in *Bombyx mori*, little is known regarding the specific *Spz* and *Toll* genes members involved in innate immunity. There is also limited direct evidence of the interaction between Spz and Toll. In this study, the dual-luciferase reporter assay results showed that BmToll11 and BmToll9–1 could activate both *drosomycin* and *diptericin* promoters in S2 cells. Furthermore, *BmToll11*, *BmToll9–1*, and five *BmSpzs* genes were found to be significantly upregulated in *B. mori* infected by *Escherichia coli* and *Staphylococcus aureus*. Additionally, the yeast two-hybrid assay results confirmed that BmSpz2, but not other BmSpzs, could interact with both BmToll11 and BmToll9–1. These findings suggest that the activated BmSpz2 can bind with BmToll11 and BmToll9–1 to induce the expression of AMPs after the silkworm is infected by pathogens.

## 1. Introduction

Innate immunity works as an evolutionarily conserved system, which provides the first defense line in animals against a wide variety of pathogens [1]. Antimicrobial peptides (AMPs) are an important family of innate immunity effectors in insects, they are involved in the three main signaling transduction pathways—Toll, JAK/STAT, and immune deficiency (IMD) [2]. The critical step of innate immune responses in the Toll signaling pathway is that pathogen-associated molecular patterns (PAMPs) are recognized by host pattern recognition receptors (PRRs) [1,3]. Toll-like receptors (TLRs) are composed of extracellular leucine-rich repeat (LRR) arrays and an intracellular Toll-interleukin-1 receptor (TIR) domain and are a group of PRRs that can recognize specific PAMPs. In insects, PAMP recognition by TLRs results in the activation of AMPs, which subsequently involves the innate immune response [4].

Among the nine Toll genes identified in *Drosophila melanogaster*, the first *Toll* gene was reported for its function in patterning during development [5]. Later, it was confirmed that *D. melanogaster* TLRs also participate in innate immune response [6,7]. *Drosophila* Toll signaling pathways are activated by Gram-positive bacteria, yeast, or fungi but not Gram-negative bacteria [8,9]. When pathogens infect *Drosophila*, PG recognition proteins (PGRPs) activate serine protease zymogens, resulting in the cleavage of pro-Spätzle (proSpz) [10,11]. During this process, the Spz-processing enzyme (SPE) is specifically activated and cleaves proSpz into the mature active C-terminal C-106 domain [12,13,14]. Then, the activated Spzs bind to the extracellular region of the Toll receptor [12,15,16]. After the Spz binding to the Toll receptor, the conformation of Toll is changed. Then, an active form of the Toll dimer is generated [17]. Once activated, the TIR domain of Toll forms a homotypic interaction with MyD88 [18,19]. MyD88, Tube and Pelle bind together to form a signaling complex via their death domains [20,21,22]. Then, the complex induces the translocation of their downstream transcription factors into the nucleus, resulting in the activation of AMPs [23,24].

The Toll signaling pathway is an evolutionarily and highly conserved mechanism for immune responses in insects. Following their identification in the fruit fly, *Toll* and *Spz* genes have been reported in many insects, such as *Tribolium castaneum* [25], *Anopheles gambiae* [26,27], *Apis mellifera* [28], *Manduca sexta* [29] and *Bombyx mori* [30,31]. In *M. sexta*, hemolymph proteinase 8 cleaves proSpätzle-1A to release the active C-terminal domain MsSpz-C108, a dimer of the C-terminal 108 residue cystine-knot domain [32]. Then, the binding of MsSpz-C108 and MsToll^ecto^ (the Toll ectodomain, extracellular region) activates the expression of AMPs [33].

As an important economical insect, *B. mori* has been utilized as a model insect for biological research [30,34,35]. There have been 14 *Toll* genes identified in *B. mori*, six more than in *Drosophila* [30,31,36]. Phylogenetic analyses of insect TLRs showed that six BmTLRs belong to the immunity-related group, and others were involved in various processes [30]. However, only the roles of BmToll9–1 in innate immunity have been confirmed [37,38]. As one of the ligands for the Toll, Spz can activate the Toll pathway. It has been found that there has 6, 6, 2 *Spz* genes in *Drosophila*, *Anopheles*, and *Apis*, respectively. In addition, six *BmSpz* genes have been identified in *B. mori* [39], but only *BmSpz1* and *BmSpz4* are known to involve in innate immune response [40,41]. *BmSpz3*/*Toll8* is mainly involved in melanization rather than pattern formation [39]. In addition to *Toll9–1*, there are 13 other *Toll* genes encoded in silkworms, however, whether AMP genes are activated by these members remains unknown. In addition, it is unclear which BmSpz can bind to Toll9–1 or other Tolls.

In this study, the results from S2 cells cotransfected with recombinant BmTIR plasmids and luciferase plasmids with different *D. melanogaster* promoter fragment (pGL3B-*drosomycin* or pGL3B-*diptericin*) showed that BmTIR11 and BmTIR9–1 could significantly activate both *drosomycin* and *diptericin* promoter. We further determined that BmSpz2, but not other Spzs, binds to BmToll11 and BmToll9–1. Together, our results confirmed that a Toll-Spz pathway regulated immune response to microbial infection in *B. mori*.

## 2. Materials and Methods

### 2.1. Insect Rearing, Immune Challenge, and Cell Lines

The *B. mori* strain Dazao was reared on an artificial diet (Nihon Nosanko, Kanagawa, Japan) and maintained at 25 °C under a photoperiod of 12 h light and 12 h dark.

*Escherichia coli* and *Staphylococcus aureus* were inactivated with 4% formalin for 15 min. After centrifugation at 8000× *g* for 3 min, the microbes were collected and dissolved in 0.85% sterilized NaCl. The fifth instar larvae were injected with microbes (OD600 = 0.8, 5 μL per larva), while silkworms injected with 0.85% sterilized NaCl were used as controls. The fat body was harvested at 3, 6, 12, and 24 h after injection. The collected samples were stored at −80 °C and later used to total RNA extraction.

*D. melanogaster* Schneider 2 (S2) cells were maintained in Schneider Drosophila Medium (Gibco, Waltham, MA, USA) with 10% fetal bovine serum and 1% penicillin/streptomycin (Gibco, Waltham, MA, USA) at 28 °C under a normal atmosphere in T-25 flasks (Corning, NY, USA).

BmN-SWU1, a *B. mori* cell line, was cultured in TC-100 medium (United States Biological, Salem, MA, USA) with 10% fetal bovine serum and 1% penicillin/streptomycin (Gibco, Waltham, MA, USA) and maintained at 28 °C [42].

### 2.2. RNA Isolation and cDNA Synthesis

Total RNA was extracted using a total RNA extraction kit (OMEGA, Doraville, GA, USA), and the contaminating genomic DNA was digested with RNase-free DNase I (Takara, Tokyo, Japan) for 15 min at 37 °C. Then, total RNA was used to reverse transcribe the first-strand cDNA using a commercial kit (Promega, Madison, WI, USA). The cDNA samples were stored at −80 °C.

### 2.3. Gene Cloning

All 14 *BmToll* gene sequences were obtained from the NCBI GenBank (https://www.ncbi.nlm.nih.gov/genbank), SilkDB (https://silkdb.bioinfotoolkits.net/main/species-info/-1) and KAIKObase (http://sgp.dna.affrc.go.jp/KAIKObase/) databases. The TIR domains, signal peptides, and transmembrane regions were predicted by SMART [43] (Table S1). All 14 BmTIRs were amplified by polymerase chain reaction (PCR) from cDNA samples and cloned into the pMT/BiP/V5-His A vector (V413020, Invitrogen, Waltham, MA, USA) for dual-luciferase reporter assays. Forward and reverse primers were listed in Table S2. PCR reactions were performed with the following conditions: 94 °C for 3 min, 35 cycles of 94 °C for 30 s, Tm−5 °C for 30 s and 72 °C for 1 min, then a final extension at 72 °C for 10 min. The PCR products were purified using a gel purification kit (OMEGA, Doraville, GA, USA). The purified PCR products were first inserted into the pMD-19T vector (Takara, Tokyo, Japan). Recombinant plasmids were purified using a Pure Yield™ Plasmid Miniprep System (A1222, Promega, Madison, WI, USA) and digested with respective restriction enzymes. The purified DNA fragments were inserted into the pMT/BiP/V5-His A vector using T4 DNA ligase (M0202L, NEB, Beverly, MA, USA). Positive clones were sequenced by the Sangon Company (Shanghai, China) for further experiments.

The construction of recombinant plasmids for the study of BmToll11 and Toll9–1 involved in the immune response and yeast two-hybrid assays was performed as described above. For the analysis of immune responses, *BmTIR11*, *BmTIR9–1*, *BmSpz2* (the active domain), and *DsRed* (as a control) genes were inserted into the pIZT/V5-His vector (V8010, Invitrogen, Waltham, MA, USA). For the yeast two-hybrid assay, *BmToll^ecto^11* and *BmToll^ecto^ 9–1* were cloned into the pGBKT7 vector (Takara, Tokyo, Japan), while the active domain of *BmSpzs* was cloned into the pGADT7 vector (Takara, Tokyo, Japan). The mature active C-terminal domain of BmSpzs was cleaved by pro-Spätzle (proSpz), and the predicted cleavage sites were listed in Figure S2. The forward and reverse primers were listed in Table S2.

### 2.4. Dual-Luciferase Reporter Assay

For the dual-luciferase reporter assay, S2 cells were plated in 6-well culture plates (10^5^ cells/well) and incubated overnight in serum-free medium. Then, S2 cells were transiently cotransfected with recombinant pMT-TIR expression plasmids (2 µg), pGL3B, pGL3B-*drosomycin* or pGL3B-*diptericin* firefly luciferase reporter plasmids (1 µg) and Renilla luciferase reporter plasmid (pRL-TK, Promega, Madison, WI, USA) (0.1 µg) [33,44]. After overnight transfection, serum-free medium was replaced with complete growth medium containing 250 mM copper sulfate for protein expression. Then, firefly luciferase and Renilla luciferase activities were measured at 48 h after the induction of protein expression using the Dual-Luciferase Reporter Assay System (E1960, Promega, Madison, WI, USA) in the GloMax^®^-Multi+ Detection System (Promega, Madison, WI, USA). Relative luciferase activity (RLA) was obtained as the ratio of firefly luciferase activity to Renilla luciferase activity. RLA from S2 cells cotransfected with empty pMT/BiP/V5-His A and pGL3B (empty reporter vector) plasmids were used as the calibrator.

### 2.5. Synthesis of dsRNA

The interference segments of *BmToll11*, *BmToll9-1*, *BmSpz2*, and *EGFP* (control) genes were designed by the database (http://sidirect2.rnai.jp/). T7 promoter sequences were tailed to sense and antisense primers (primers sequences in Table S2). Synthesis of dsRNA were used with Transcript Aid T7 High Yield Transcription Kit (KO441, Thermo Scientific, Waltham, MA, USA), which were purified using MicroElute RNA Clean-up Kit (R6247, OMEGA, Doraville, GA, USA) for RNA interference (RNAi).

### 2.6. Immune Response Analysis

For immune response analysis, the *B. mori* cell line BmN-SWU1 was plated in 6-well culture plates (10^5^ cells/well) and incubated overnight in serum-free medium. Then, BmN-SWU1 cells were transiently transfected with the recombinant expression plasmids pIZT/BmTIR11, pIZT/BmTIR9–1 and pIZT/BmSpz2 (2 µg), while cells cotransfected with pIZT/DsRed served as the control. After 72 h, the cells were collected for RNA isolation and cDNA synthesis, as described above.

For RNAi, the *B. mori* cell line BmN-SWU1 was plated in 6-well culture plates (10^5^ cells/well) and incubated overnight in serum-free medium. Then, BmN-SWU1 cells were transfected with the interference fragments of *BmToll11*, *BmToll9-1*, *BmSpz2*, or *EGFP* (3 µg), while cells were challenged by inactivated *E. coli* or *S. aureus* (PBS buffer were used as a control). After 48 h, the cells were collected for RNA isolation and cDNA synthesis, as described above.

### 2.7. Real-Time Quantitative PCR (RT-qPCR) Analysis

*B. mori* ribosomal protein L3 *(BmRpL3*) was used as an internal control for normalization. The 20 μL mixture included 2 μL cDNA, 0.5 μL each primer (10 mM; Table S3), 10 μL SYBR™ Select Master Mix reagent (Bio-Rad, Hercules, CA, USA) and 7 μL ddH_2_O. RT-qPCR was performed according to the following parameters: one cycle of an initial denaturation step at 95 °C for 1 min, 40 cycles at 95 °C for 30 s and 60 °C for 20 s and a final cycle at 95 °C for 15 s, 60 °C for 30 s and 95 °C for 15 s. The specific primers of genes for qPCR are listed in Table S3. The relative gene expression levels were estimated according to the 2^−ΔΔCt^ method [45]. All samples were run in triplicate.

### 2.8. Yeast Two-Hybrid Assay

Briefly, *BmTolls* and *BmSpzs* genes were fused to the yeast two-hybrid vectors pGADT7 and pGBKT7. Yeast competent cells were transformed simultaneously with the bait and prey constructs pGBKT7-BmTolls/pGADT7-BmSpzs. Yeast cells were plated on synthetic defined premix (SD) agar base plates that did not contain leucine (Leu) or tryptophan (Trp). Then, the positive clones containing the constructs pGADT7-prey/pGBKT7-bait were grown on SD plates in the absence of Leu, Trp, histidine (His), and adenine (Ade) but the presence of 5-bromo-4-chloro-3-indoxyl-D-gal (X-α-Gal).

### 2.9. Statistical Analysis

One representative set of data was used to generate figures with GraphPad Prism software (GraphPad Prism 8). All statistical analyses were conducted using SPSS software (IBM SPSS v. 22), and cumulative survival was analyzed by Tukey’s multiple comparison test. Significant differences are indicated with * for *p* < 0.05 and ** for *p* < 0.01. All results are shown as means ± SD of triplicate samples. All data presented are representative of a minimum of three independent experiments.

## 3. Results

### 3.1. Overexpression of Recombinant BmTIRs in S2 Cells Activates Drosomycin and Diptericin Reporter Genes

**Figure 1:**
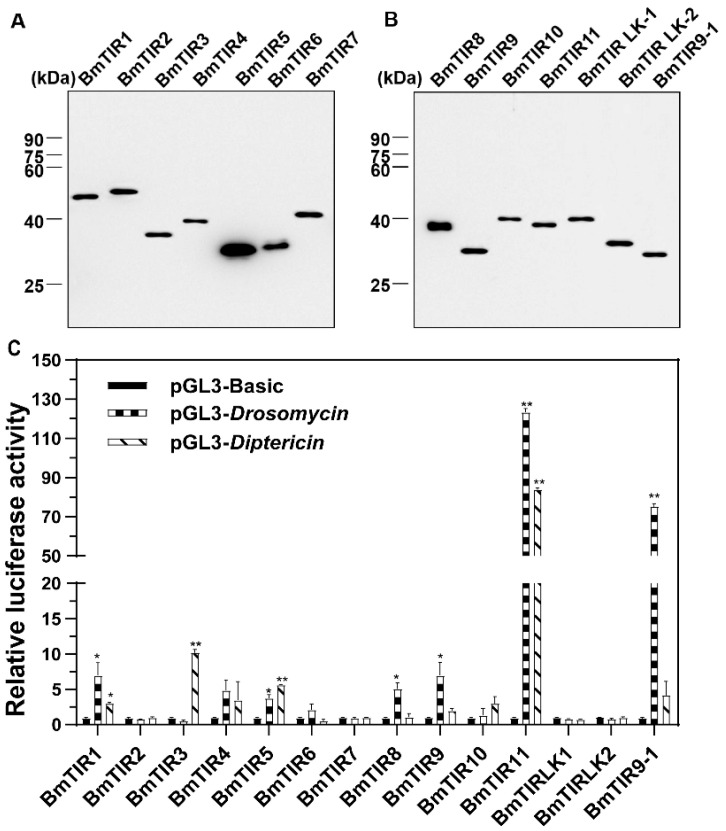
The Toll-interleukin-1 receptor (TIR) domains of *Bombyx mori* Toll family members can recognize the *drosomycin* and *diptericin* promoter. (**A**,**B**): Expression of recombinant *B. mori* TIR proteins in S2 cells. The BmTIRs expression in S2 cells were identified by Western blot analysis using an anti-V5 antibody. (**C**): Expression of BmTIRs activates the promoter activity of *drosomycin* or *diptericin* in S2 cells. The cells were cotransfected with recombinant BmTIR plasmids and luciferase plasmids with or without different promoter fragments (pGL3B, pGL3B-*drosomycin* or pGL3B-*diptericin*) for 48 h, and then the relative luciferase activity (RLA) was measured by dual luciferase assay. The statistical analysis used Tukey’s multiple comparison test. Bars represent the mean of three individual measurements ± SD. Significant differences are indicated with * for *p* < 0.05 and ** for *p* < 0.01.

The function of the Toll-Spz signaling pathway in regulating innate immunity in *D. melanogaster* is well understood. However, in other insect species, it is not well studied. In *B. mori*, *Toll* and *Spz* genes have been identified [30,31,39], but few were known that the specific Tolls and Spzs implicated in the innate immune system. To explore the Toll-Spz pathway in *B. mori*, we constructed recombinant vectors and expressed the TIR of Toll receptors (BmTIRs) in the S2 cell line. Immunoblotting results showed that recombinant BmTIR proteins were detected in S2 cells (Figure 1A,B).

We next cotransfected the different combination vectors of BmTIR proteins with the vector containing the *D. melanogaster drosomycin* or *diptericin* promoter in S2 cells, then the activity of *drosomycin* or *diptericin* promoter was measured using dual-luciferase reporter assays. The results showed that BmTIR9–1 overexpression significantly improved the levels of the relative luciferase activity (RLA) of the *drosomycin* reporter. In addition, the overexpression of *BmTIR11* gene remarkably increased the RLA of *drosomycin* and *diptericin* reporters (Figure 1C). These results showed that BmTIR11 and BmTIR9–1 could activate *drosomycin* and *diptericin* genes in S2 cells, which suggested that they may be involved in *B. mori* AMP gene regulation.

### 3.2. BmToll11 and BmToll9–1 are Involved in the Immune Response

**Figure 2:**
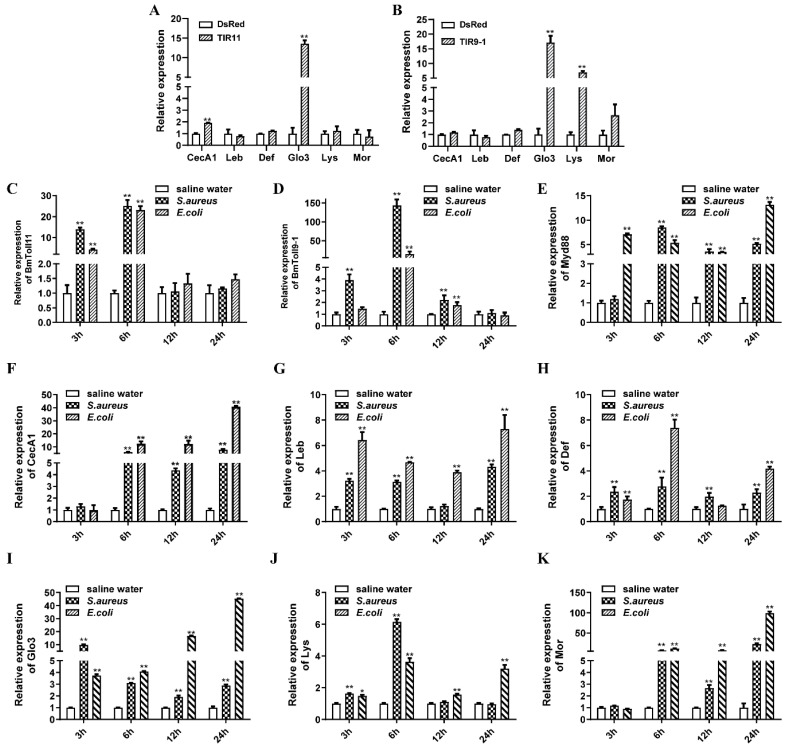
BmToll11 and BmToll9–1 activate the expression of AMPs and are involved in the immune response. (**A**,**B**): The overexpression of BmToll11 and BmToll9–1 could promote the mRNA expression of AMPs in the BmN-SWU1 cell line. BmN-SWU1 cells were transiently transfected with recombinant plasmids. The total RNAs were prepared after transfected for 72 h, cDNA was synthesized with olig (dT)-primer. Then the AMPs were determined by RT-qPCR. (**C**–**K**): The Toll signaling pathway was activated by *Escherichia coli* or *Staphylococcus aureus*. The fifth instar larvae were injected with *E. coli* or *S. aureus*, while the 0.85% sterilized NaCl was used as control. The fat body was harvested for the cDNA synthesis at 3, 6, 12, and 24 h after injection. Then *BmToll11*, *BmToll9–1*, *Myd88*, and AMPs were detected by RT-qPCR. For all RT-qPCR, *RPL3* was used as an internal control. Bars represent the mean of three individual measurements ± SD. Significant differences are indicated with * for *p* < 0.05 and ** for *p* < 0.01, which is determined by one-way ANOVA followed by a Tukey’s multiple comparison test.

To investigate whether *B. mori Toll11* and *Toll9–1* genes were involved in the immune response in silkworms, the mRNA expression levels of *B. mori* AMP genes were analyzed by RT-qPCR in BmN-SWU1 cells overexpressing BmTIR11 or BmTIR9–1. First of all, overexpression levels of *BmTIR11* or *BmTIR9–1* genes were tested, which results in a significant up-regulation in BmN-SWU1 cells overexpressing BmTIRs (Figure S3A). *Cecropin A1* and *gloverin-like protein 3* genes were upregulated in BmN-SWU1 cells overexpressing *BmTIR11* (Figure 2A). *Gloverin-like protein 3* and *lysozyme* genes were upregulated in BmN-SWU1 cells overexpressing *BmTIR9–1* (Figure 2B). Then, the expression levels of *B. mori* AMP genes were also analyzed in BmN-SWU1 cells knocking down *BmTIR11* or *BmTIR9–1* genes. The expressions of AMPs were inhibited in *BmToll11* or *BmToll9–1* gene knockdown cells (Figure S4A,B). These results showed that *BmToll11* and *BmToll9–1* could activate the immune response in the BmN-SWU1 cell line. Furthermore, an experiment was performed using silkworms infected with *E. coli* (Gram-negative bacteria) or *S. aureus* (Gram-positive bacteria). Then, the transcriptional levels of *B. mori Toll11* and *Toll9–1* genes were analyzed by RT-qPCR at different times after infection. The RT-qPCR results showed that the *Toll11* and *Toll9–1* genes of *B. mori* were activated by microbes at 3 h and 6 h compared with saline water but downregulated after 12 h (Figure 2C,D). The *Myd88* was also activated by the two microbes (Figure 2E). These results suggested that both Gram-negative and Gram-positive bacteria could induce the expression of *BmToll11* and *BmToll9–1* genes.

In *D. melanogaster*, some AMP genes expression was regulated through the Toll pathway activated by fungi or Gram-positive bacteria, such as *drosomycins*, *metchnikowin*, and *defensins*. Another AMP genes expression was regulated by the IMD pathway activated by Gram-negative bacteria, such as *drosocin*, *diptericin*, and *attacins*. Several AMP genes in silkworms, including *cecropin A1* (CecA1), *lebocin* (Leb), *defensins* (Def), *gloverin-like protein 3* (Glo3), *lysozyme* (Lys), and *moricin 1* (Mor) were activated by the two microbes (Figure 2F–K). These results suggest that both Gram-negative and Gram-positive bacteria could activate the Toll signaling pathways and cause the increased expression of *BmToll11*, *BmToll9–1*, and AMPs in silkworms.

### 3.3. BmToll^ecto^9–1 and BmToll^ecto^11 Interact with BmSpz2 but not Other BmSpzs

**Figure 3:**
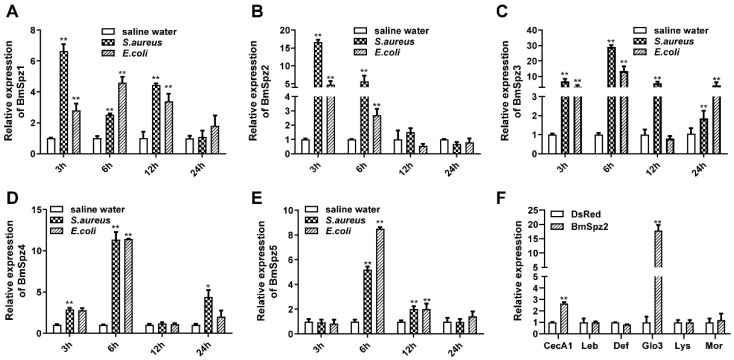
BmSpzs were activated by *E. coli* or *S. aureus* in *B. mori* larvae, while AMPs were activated in BmSpz2 overexpressed cells. (**A**–**E**): RT-qPCR was performed to detect the mRNA expression level of BmSpzs of silkworm challenged by two microbes respectively. (**F**): The AMPs were detected by RT-qPCR in BmSpz2 overexpressed cells. *RPL3* was used as an internal control. Bars represent the mean of three individual measurements ± SD. Significant differences are indicated with * for *p* < 0.05 and ** for *p* < 0.01, which is determined by one-way ANOVA followed by a Tukey’s multiple comparison test.

The mechanism that the activation of the Toll signaling pathway in *D. melanogaster* and *M. sexta* is relatively clear. In *Drosophila*, the Toll pathway is activated after the activated Spz binding with the Toll receptor [6]. We demonstrated that *BmToll11* and *BmToll9–1* genes function in innate immune responses in silkworms. However, it is unclear which Spzs may be involved in innate immune responses and interact with BmTolls. The amino acid sequence of Spz contains a cystine knot domain (Spz CK domain) (Figure S2). Furthermore, phylogenetic analysis was conducted using the CK domain sequence of *Spz* genes, the results showed that there was a highly conserved monophyly among different insect species (Figure S1). To identify which of the five *BmSpzs* genes may be involved in innate immune responses, RT-qPCR was performed. The results showed that all five *BmSpzs* genes were upregulated after the injection of inactivated bacteria for 6 h (Figure 3).

**Figure 4:**
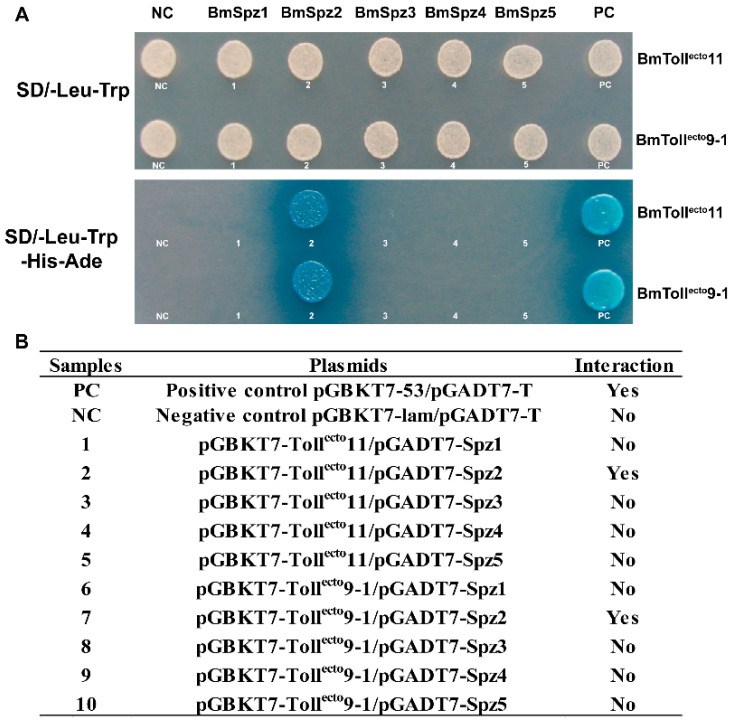
BmToll^ecto^11 and BmToll^ecto^9–1 interact with BmSpz2 but not other BmSpzs. (**A**): Yeast two-hybrid assay for determination of BmToll^ecto^11 and BmToll^ecto^ 9–1 with BmSpz2. Yeast cells were transformed simultaneously with the bait (pGBKT7-BmToll^ecto^s) and prey constructs (pGADT7-BmSpzs). On the up line, yeast cells containing the bait and prey constructs were plated on synthetic defined premix (SD) agar base plates that did not contain leucine (Leu) or tryptophan (Trp). Howere, on the down line, only the clones containing the constructs pGBKT7-53/pGADT7-T (PC), pGADT7-Toll11^ecto^/pGBKT7-Spz2 and pGADT7-Toll9-1^ecto^/pGBKT7-Spz2 could grow on SD plates in the absence of Leu, Trp, histidine (His), and adenine (Ade). These clones also formed blue colonies in the presence of 5-bromo-4-chloro-3-indoxyl-D-gal (X-α-Gal). (**B**): The table summarized the information given above A. BmToll^ecto^11 and BmToll^ecto^9–1 interact with BmSpz2, while other BmSpzs were not. Positive control pGBKT7-53/pGADT7-T (PC) and negative control pGBKT7-lam/pGADT7-T (NC) reactions were provided for each group.

To determine which Spzs interact with Toll11 and Toll9–1, a yeast two-hybrid analysis was conducted. The results confirmed that BmSpz2 could interact with both BmToll^ecto^11 and BmToll^ecto^9–1, whereas other BmSpzs failed to interact with them (Figure 4). Furthermore, the mRNA expression levels of *B. mori AMP* genes were analyzed by RT-qPCR in BmN-SWU1 cells overexpressing BmSpz2. *Cecropin A1* and *gloverin-like protein 3* genes were upregulated in BmN-SWU1 cells overexpressing BmSpz2 (Figure 3F). The expression levels of *B. mori* AMP genes were also analyzed in BmSpz2 knocking down BmN-SWU1 cells. The expressions of AMPs were inhibited in *BmSpz2* gene knocking down cells (Figure S4C). These results suggested that BmToll11 and BmToll9–1 could bind to BmSpz2 and activate the expression of AMPs in *B. mori*.

## 4. Discussion

In insects, the Toll-Spätzle pathway works as an evolutionarily conserved system, which plays a key role in their innate immunity against pathogens. In *D. melanogaster*, Toll1 and Spz1 were implicated in regulating AMP genes and other immune-related functions [9,46]. In *B. mori*, BmSpz1, BmSpz4 and BmToll9–1 have been confirmed to activate the Toll signaling pathway and function in innate immunity [37,38,40,41]. In addition to Spz1, Spz4, and BmToll9–1, whether the other Tolls and Spzs can activate innate immunity pathways are unknown. The results of our dual-luciferase reporter assays showed that BmTIR11 and BmTIR9–1 were strongly elicited AMP genes (Figure 1C). The findings also confirmed that *BmToll11* and *BmToll9-1* genes were upregulated when silkworms were challenged with *E. coli* or *S. aureus* (Figure 2C,D) and that they could activate the transcription of AMP genes (Figure 2). In vivo experiments showed that the transcriptional expression levels of five Spzs were all increased in the silkworm (Figure 3). These results showed that BmToll9–1, BmToll11, and BmSpz1–5 were involved in the innate immunity in silkworms.

Although the interactions between Tolls and Spzs were well studied in *D. melanogaster* and *M. sexta* [6,33,46], it remains unclear whether the Tolls can participate in the innate immune response in the silkworm through binding with BmSpz1 or other Spzs. The yeast two-hybrid assay results showed that BmToll11 and BmToll9-1 could interact with BmSpz2 and activate the expression of AMP genes (Figure 2 and Figure 4). Additionally, the results indicated that one BmSpz could combine with multiple BmTolls, which is consistent with the reported binding of Spzs to multiple Tolls in *D. melanogaster* [6]. In our study, we also found that other Tolls, except for BmToll9–1 and BmToll11, participated in immune regulation, including BmToll 1, BmToll 3, BmToll 5, and others (Figure 1B). Five *BmSpzs* genes were also upregulated when the silkworm was challenged by two microbes (Figure 3). Therefore, we speculate that except for BmSpz2, the other Spzs can also bind to other BmTolls. Furthermore, it might be possible that multiple BmSpzs can bind to one or more BmTolls. However, further studies are required to confirm these predictions.

Insects lack an acquired immune system and mainly rely on innate immunity to resist pathogens infection. AMPs play an important defense role in insect innate immunity that are regulated by many signals, such as the Toll and IMD signal transduction pathways [3,7,47,48]. In *D. melanogaster*, seven kinds of AMPs are synthesized. Some of the AMPs genes expression was regulated by the Toll pathway activated by fungi or Gram-positive bacteria, such as *drosomycins*, *metchnikowin*, and *defensins*, whereas another AMPs expression regulated by the IMD pathway activated by Gram-negative bacteria, such as *cecropins*, *drosocin*, *diptericin*, and *attacins*. In *M. sexta*, AMP genes, including *attacin*, *cecropin*, *lysozyme gloverin*, *lebocin*, and *moricin* have been identified [49,50]. Among these genes, *attacin* and *cecropin* are widely found in most insect species, whereas *lebocin*, *moricin*, and *gloverin* were conserved in Lepidoptera species [49,51,52,53]. These genes can be activated by the host infected by different bacterial [54]. The overexpression of MsTIR and DmTIR in S2 cells can activate *drosomycin* (a target gene of the Toll pathway) but not *diptericin* (a target gene of the IMD pathway) [33]. At present, the members of *B. mori* AMPs can be classified into six different families, including *cecropin*, *attacin*, *moricin*, *gloverin*, *lebocin*, and *defensin* [35,51,55,56,57,58,59]. The activation of AMPs is different depending on the pathogen used to challenge silkworms [38,41,60]. Our studies showed that the Toll pathways were both activated by Gram-positive and Gram-negative bacteria in *B. mori* (Figure 1 and Figure 2), unlike in *M. sexta* or *D. melanogaster* [6,33], which is consistent with a recent study in *Tenebrio molitor* [61]. In addition, the activation of AMP genes was not absolutely specific following the challenge with *E. coli* or *S. aureus* (Figure 2). Our results indicated that the activation of the Toll receptor signaling pathway and the regulation of AMP gene expression are different between *B. mori* and *D. melanogaster*.

## 5. Conclusions

In summary, *B. mori* infected by pathogens could activate BmSpz2 to bind with BmToll11 and BmToll9–1, and then induce the expression of AMPs. In this study, we identified a Toll-Spz pathway involved in the immune response to bacteria in *B. mori*. The findings of this study may help improve the understanding of the signaling pathways involved in Lepidoptera insect innate immune responses.

## Supplementary Materials

The following are available online at https://www.mdpi.com/2075-4450/11/9/586/s1, Figure S1, A phylogenic tree of insect Spzs. Figure S2, Alignment of the amino acid sequences of the Spz cystine-knot domains in *D. melanogaster*, *M. sexta*, and *B. mori*. Figure S3, The evaluation of BmToll11, BmToll9–1 and BmSpz2 expression by RT-qPCR in gene overexpression or knock-down cells. Figure S4. The expressions of AMPs were inhibited in BmToll11, BmToll9–1 and BmSpz2 knock down cells. Table S1, Features of BmToll genes of *B. mori*. Table S2, Oligonucleotide primers.
