# Supplementary material for: A Toll-Spätzle Pathway in the Immune Response of Bombyx mori
Source: Insects. 2020 Sep 1;11(9):586. doi: 10.3390/insects11090586 (PMC7564906; doi:10.3390/insects11090586)
Supplement: Supplementary file 1 [file insects-11-00586-s001.pdf]

Supplemental Data

Fig. S1.

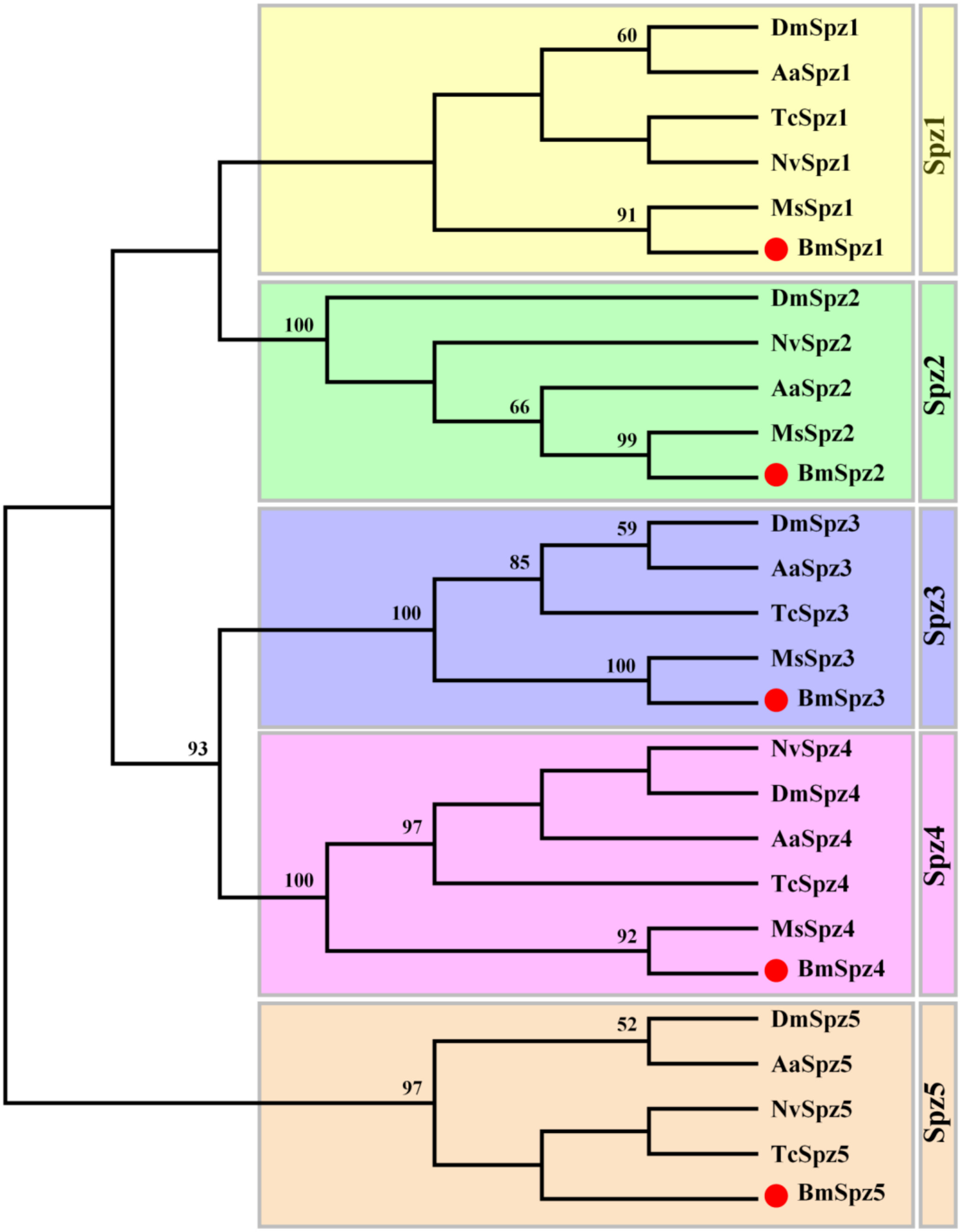

Fig. S1. A phylogenic tree of insect Spzs. The protein sequences of CK domain were used as queries for searching homologs of BmSpz genes with the tBLASTN program from SilkDB. To identify orthologous relationships among spz family genes in insects, phylogenetic analysis was conducted using the CK domain sequence of genes, which was predicted by Pfam (pfam.xfam.org/). Accession numbers on spz family genes in *B. mori* (Bmspz1: BGIBMGA002397, Bmspz2: BGIBMGA010871, Bmspz3: XP_012550687.1, Bmspz4: BGIBMGA008841, Bmspz5: BGIBMGA012697), *D. elanogaster* (DmSpz1: AAF56658.1, DmSpz2:NP_729009.2, DmSpz3: AAF52574.2, DmSpz4: NP_609504.2, DmSpz5:AAF47694.1), *M.sexta* (MsSpz1:ACU68553.1, MsSpz2: Msex2.09986-PA, MsSpz3: Msex2.04433-PA, MsSpz4:Msex2.03391-PA), *T. castaneum* (TcSpz1: EEZ99207.1, TcSpz3: NP_001153625.1, TcSpz4: EFA09263.1, TcSpz5: XP_970793.1, TcSpz6: NP_001164082.1), *A. aegypti*

(AaSpz1: EAT34304.1, AaSpz2: EAT47471.1, AaSpz3: EAT39601, AaSpz4: XP_001652981.2, AaSpz5: XP_001654338.1) and *N. vitripennis* (NvSpz1: XP_001606369, NvSpz2: XP_001607462, NvSpz4: XP_001605307, NvSpz5: XP_001599503) were obtained from the NCBI GenBank (https://www.ncbi.nlm.nih.gov/genbank), SilkDB (https://silkdb.bioinfotoolkits.net/main/species-info/-1), KAIKObase (sgp.dna.affrc.go.jp/KAIKObase/) and InsectBase (http://www.insect-genome.com/) public databases. Amino acid sequence alignment was performed with the multiple sequence alignment by Clustal W (http://clustalw.ddbj.nig.ac.jp/top-e.html). Phylogenetic trees were constructed by the neighbor-joining method with a Poisson correction model, using mega version 7.0.26. For the neighbor-joining method, gaps were treated as characters, and statistical analysis was performed by the bootstrap method, using 1000 repetitions.

Fig. S2.

```
                                                  1                                  2
DmSpz1  ----FSNDLQPTDVSSRVGGSDE--RFLCRSIRKLVYPKKGLRADDTWQLIVN-NDEYKQAIQIEEC  60
MsSpz1  ----ELDVSDTPDIAQRLGPQEDN-MELCSFREKIFYPKAAPDKDGNWFFVVNSKENPVQGYKVEIC  62
BmSpz1  ----KLDLPSIPDIAQRAGSFEDS-YELCDFRVQIMTPLAAQSDDLKWYHVLNFNENPLQGFRVEIC  62
BmSpz2  ----YLVSDRRGDTANRDFASAGEAGFMCPSTVKYARPQRARATSGHWKYIVN-TGEHTQTLRLEKC  62
BmSpz4  ----EVRVKPQHQEGSRPAVIKLGGANACESTETLTAPFWANSTRGEVLALLN-MHPFEQYIHMETC  62
BmSpz5  ----ALGLASAETSAERPRRQAVNAQELCSVRTEYITPRAALNNKGNWKYVVNMPDNMTQLVRAEIC  63
BmSpz3  ---RHARQASNTQLPDAQAVNSTGRVDACESKTEIMTPYWALNSARKLRAIVN-TMHFEQAIHQETC  63

                 3             1                              42 3
DmSpz1  EGADQP--CDFAANFPQSYNPICKQHYTQQTLASIKSDGE-LDVVQNSFKIPSCCKCALKTG---  119
MsSpz1  DRQQLP--CAEFASFQQGYEARCIQKYVRRTMLALDPKG---QMTDMPLKVPSCCSCVAKLTII-  121
BmSpz1  NTTSTG--CAKFVTMENNYNPKCVQKFIFRKMKILSESG---EMIERSMKVPSCCSCVATLL---  119
BmSpz2  LKPKDS--CTYLTDNFQSK---CVQVYNYHRLLTWDQQN---GLHMDIFKVPTCCSCHI------  113
BmSpz4  LHERKQMYCREGCR--------CEQQYRLHRLLAYDPRNECRGIFADWFRFPTCCVCKCYDVPVE  119
BmSpz5  TSTT----CSGLCTIPSGYTSRCEQKYIQKRLVALQSSGQ--NLYTDIFWIPSCCQCSIIP----  118
BmSpz3  SKTTTGR-CNRDCG--------CEQKYKWHRLLAYDPSDDCAGIFMDWFLFPSCCVCRCKP----  115
```

Fig. S2. Alignment of the amino acid sequences of the Spz cystine-knot domains in *D. melanogaster*, *M. sexta* and *B. mori*. The proteolytic activation site is right after R (bolded and underlined) and the scissile bond is indicated with asterisks. Absolutely conserved cysteines are shaded and numbered. The paired numbers (1–1, 2–2, 3–3) indicate the intrachain disulfide linkage in DmSpz. Cys4 forms an intermolecular disulfide bond with its counterpart in another subunit.

Fig. S3.

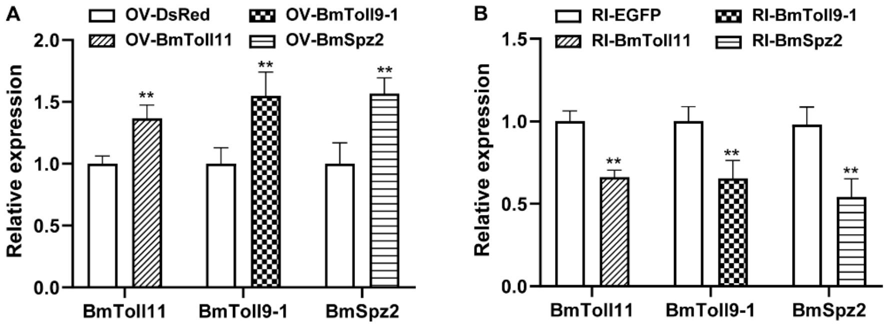

Fig. S3. The evaluation of BmToll11, BmToll9–1 and BmSpz2 expression by RT-qPCR in gene overexpression or knock-down cells. A: The mRNA expression of BmToll11, BmToll9–1 and BmSpz2 was significantly up-regulated in their own overexpressing cells respectively. OV-BmToll11, OV-BmToll9–1, OV-BmSpz2 and OV-DsRed represents cells transfected with the recombinant expression plasmids pIZT/BmTIR11, pIZT/BmTIR9–1, pIZT/BmSpz2 and pIZT/ DsRed respectively. B: The expression levels of BmToll11, BmToll9–1 and BmSpz2 were decreased obviously in gene knock-down cells. RI-BmToll11, RI-BmToll9–1, RI-BmSpz2 and RI-EGFP indicates BmN-SWU1 cells transfected with BmToll11 siRNA, BmToll9–1 siRNA, BmSpz2 siRNA and EGFP siRNA, respectively. *RPL3* was used as an internal control. Bars represent the mean of three individual measurements ± SD. Significant differences are indicated with * for P < 0.05 and ** for P < 0.01, which determined by one way ANOVA followed by a Tukey's multiple comparison test.

Fig. S4.

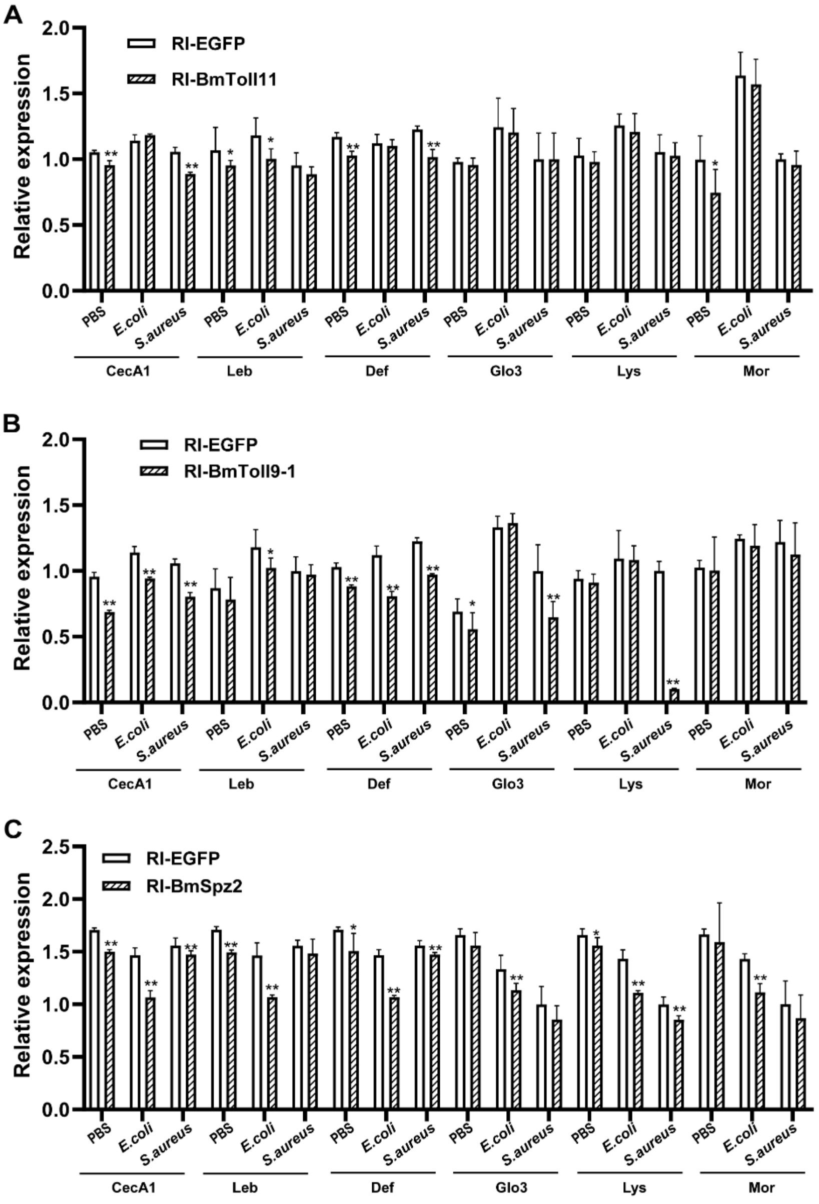

Fig. S4. The expressions of AMPs were inhibited in BmToll11, BmToll9–1 and BmSpz2 knock down cells. RI-BmToll11, RI-BmToll9–1, RI-BmSpz2 and RI-EGFP indicates BmN-SWU1 cells transfected with BmToll11 siRNA （A）, BmToll9–1 siRNA （B）, BmSpz2 siRNA （C) and EGFP siRNA, respectively. Then inactivated pathogens,

including *E.coli* and *S.aureus* were added into BmN-SWU1 cells. The AMPs were determined by RT-qPCR. The results showed the expressions of BmToll11, BmToll9–1 and BmSpz2 were decreased obviously in knock down cells. *RPL3* gene expression was used as an internal control. The Bars represent the mean of three individual measurements ± SD. Significant differences are indicated with * for $P < 0.05$ and ** for $P < 0.01$, which determined by one way ANOVA followed by a Tukey's multiple comparison test.

Table S1. Features of BmToll genes of *B.mori*

BmTolls	Gene ID	Size	TIR domain	Signal peptides	transmembrane regions
BmToll 1	BGIBMGA011037	1295aa	1024-1166	1-17	973-995
BmToll 2	BGIBMGA011038	1283aa	1023-1165	1-19	972-994
BmToll 3	BGIBMGA014373	614aa	450-585	NO	395-417
BmToll 4	BGIBMGA014370	1018aa	892-1027	1-19	840-862
BmToll 5	BGIBMGA010304	728aa	540-678	NO	488-510
BmToll 6	BGIBMGA011084	1275aa	1074-1211	1-29	1020-1042
BmToll 7	XP_004921732.1	1251aa	1025-1163	1-20	975-997
BmToll 8	BGIBMGA011085	1275aa	1071-1206	1-21	1019-1041
BmToll 9	BGIBMGA008840	825aa	681-824	1-19	624-646
BmToll 10	BGIBMGA011082	1188aa	918-1059	NO	863-885
BmToll 11	BGIBMGA011025	1199aa	915-1057	NO	859-881
BmToll–LK1	XP_012543930.1	1365aa	1043-1172	NO	971-993
BmToll–LK2	BGIBMGA006244	797aa	616-739	1-16	565-587
BmToll 9-1	BGIBMGA011216	755aa	603-744	1-21	551-570

Table S2. Oligonucleotide primers

Target gene and name	Sequence (5′ → 3′)
The vectors construction for recombinant BmTIRs	
BmToll1	
> BmTIR1-F-*Bgl* II	CGGAagatctTTTGAGGAGAATCAAAAATTA
> BmTIR1-R-*Xho* I	CCGctcgagGACTAGGTAGGCTTGCACGGCGTT
BmToll2	
> BmTIR2-F-*Bgl* II	GGAagatctGCGTACGACGACACTGATAAATTA
> BmTIR2 -R-*Xho* I	CCGctcgagGACAAGATAAGCTTGACCTGA
BmToll3	
> BmTIR3-F-*Bgl* II	GGAagatctGAGCCGGACGACGGGCGTCGG
>BmTIR3-R- *Eco*R V	GGAgatatcCTGCGGGCGTGGCGCTACTTGTAG
BmToll4	

> BmTIR4-F-*Bgl* II	GGAagatctGAGCCGGACGACGGGCGTCGG
>BmTIR4- *Eco*R V	GGAgatatcCGTGTCCGTCTCGGCTTCGGGCGA
BmToll5	
> BmTIR5-F-*Bgl* II	GGAagatctGAGCTAGACAAGGACAAAAGA
> BmTIR5-R-*Xho* I	CCGctcgagTAATTCAGCATTCGCTGGTGT
BmToll6	
> BmTIR6-F-*Bgl* II	GGAagatctGAAGAAGCCGACAAAGATCGCCTT
> BmTIR6-R-*Xho* I	CCGctcgagGCCCGGCGGTGGTGTTGGGGG
BmToll7	
> BmTIR7-F-*Bgl* II	GGAagatctGCCTTTAAGGATACCGATAAACTT
> BmTIR7-R-*Xho* I	CCGctcgagTACCAGGTACGTTTGAACAGT
BmToll8	
> BmTIR8-F-*Eco*R V	GGCgatatcGACTTGGAGGATTCGGAAAAAATG
> BmTIR8-R-*Xho* I	CCGctcgagCACATGGACGGACATGGTGCG
BmToll9	
> BmTIR9-F-*Bgl* II	GGAagatctCATGTAGAGACAAAGGATTACAAG
> BmTIR9-R-*Xho* I	CCGctcgagTATCTTCCGTGTCAGTACATT
BmToll10	
> BmTIR10-F-*Bgl* II	GGAagatctCTTAACGACAGCAAAAACAAACGG
> BmTIR10-R-*Xho* I	CCGctcgagAACGAAATATGTCTTTCTTGT
BmToll11	
> BmTIR11-F-*Bgl* II	GGAagatctAGCGAACTCGATAGGGATAGGTTA
> BmTIR11-R-*Xho* I	CCGctcgagTCTGTGCAAGACTGAATGAGT
BmToll–LK1	
> BmTIR–LK1-F-*Bgl* II	GGAagatctGACTATTACGTGATATGCAATACC
> BmTIR–LK1-R-*Xho* I	CCGctcgagAACAAAGTATGTGCGTCCCTT
BmToll–LK2	
> BmTIR–LK2-F-*Bgl* II	GGAagatctAAAAAGGGCATACGGATTATCGAA
> BmTIR–LK2-R-*Xho* I	CCGctcgagAACTAGACTCTGGTCTAGAAT
BmToll9-1	
> BmTIR9-1-F-*Bgl* II	GGAagatctGGTGTCGATGGTACAATATTCAAC
> BmTIR9-1-R-*Xho* I	CCGctcgagAGCTAAAGATACGTTTTCCGT

The vectors construction for BmToll11 and BmToll9–1 involving in the immune response

BmToll 11	
> BmTIR 11-F-*Bam*H I	GGAggatccATGAGCGAACTCGATAGGGATAGGTTA
> BmTIR11-R-*Xho* I	CCGctcgagTCTGTGCAAGACTGAATGAGT
BmToll 9-1	
> BmTIR 9-1-F-*Bam*H I	GGAggatccATGGGTGTCGATGGTACAATATTCAAC
> BmTIR 9-1-R-*Xho* I	CCGctcgagAGCTAAAGATACGTTTTCCGT
BmSpz2	
> BmSpz2-F1-*Bam*H I	GGAggatccATGGACTTCGCCAGCGCCGGCGA
> BmSpz2-R3-*Xho* I	TCGGctcgagCTTAAAGTTTCTTTCTATCAACT

The vectors construction for yeast two-hybrid assay

BmSpz1	
> BmSpz1-F-*Nde* I	CGCcatatgGCAGGCTCATTCGAAGACTC
> BmSpz1-R-*Bam*H I	CGCggatccCTTAACCGAGTAGCGTGGCAAC
BmSpz2	
> BmSpz2-F-*Eco*R I	CCGgaattcGACTTCGCCAGCGCCGGCGA
> BmSpz2-R-*Xho* I	CCGctcgagCTTAAAGTTTCTTTCTATCAACT
BmSpz3	
>BmSpz3-F-*Nde* I	CGCcatatgTCAGGCCGTCAACAGTACCGGA
> BmSpz3-R-*Bam*H I	CGCggatccCTCAAGGCTTACACCTGCAAACGCA
BmSpz4	
> BmSpz4-F-*Nde* I	CGCcatatgTCCTGCAGTCATTAAGCTTGGC
> BmSpz4-R-*Bam*H I	CGCggatccCTTA AGGCGATCGAGCTCGGAACTC
BmSpz5	
> BmSpz5-F-*Nde* I	CGCcatatgCAGGCTGTAAATGCTCAAG
> BmSpz5-R-*Bam*H I	CGCggatccTTAATTATTAGGTATAATGC
BmToll11	
>BmToll^{ecto}11-F-*Sma* I	TCCcccgggAATGACCATGGAATTTCACGCCGAA
>BmToll^{ecto}11-R-*Pst* I	AActgcagTTAGACTAGGTAGGCTTGCACGGCGTT
BmToll9-1	
>BmToll^{ecto}9-1-F-*Nde*I	CGCcatatgAGGAAAACACAAAAATGCTTAACC
>BmToll^{ecto}9-1-R- *Bam*H I	CGCggatccTTATTTGTACTGATTTTTAATCATCTT

The Oligonucleotide primers for synthesis of dsRNA

BmToll11	
>RI-Toll11-T7-F	***TAATACGACTCACTATAGGGAGA***AGCGAACTCGATAGGGATAG
>RI-Toll11-T7-R	***TAATACGACTCACTATAGGGAGA***ATTACATGTTTGATTGCCGC
BmToll9-1	
>RI-Toll9-1-T7-F	***TAATACGACTCACTATAGGGAGA***GGTGTCGATGGTACAATATTC
>RI-Toll9-1-T7-R	***TAATACGACTCACTATAGGGAGA***TCTAACAGCCTGTGCTGGGC
BmSpz2	
>RI-Spz2-T7-F	***TAATACGACTCACTATAGGGAGA***GCTGGGTTCATGTGTCCCAGC
>RI-Spz2-T7-R	***TAATACGACTCACTATAGGGAGA***ACCGAGGGGCGGGAACGAGA
EGFP	
>RI-EGFP-T7-F	***TAATACGACTCACTATAGGGAGA***GGTGCCCATCCTGGTCGAGCT
>RI-EGFP-T7-R	***TAATACGACTCACTATAGGGAGA***GCTGGTAGTGGTCGGCGAGCT

The Oligonucleotide primers for RT-qPCR

BmToll 11	
>Toll 11-qPCR-F	TCTTAGATCGTTGGAGGTT
>Toll 11-qPCR-R	GATTGAAATAGTTCTGGAGGT
BmToll 9-1	
>Toll 9-1-qPCR-F	GAGTCAGTGGTGCCAGTTC
>Toll 9-1-qPCR-R	CAGATAGTGGAGGGTCGTT

BmSpz1	
>Spz1-qPCR-F	ACTGGCAGGTCAATCGGATG
>Spz1-qPCR-R	CATTTCGCCGCTTTCGCTTA
BmSpz2	
>Spz2-qPCR-F	ACACAGGGGAACACACACAG
>Spz2-qPCR-R	GTCCTCACCGGGGAAATGTT
BmSpz3	
>Spz3-qPCR-F	CAGTACCGGAAGGGTGGATG
>Spz3-qPCR-R	GTCGTGGTTTTGCTGCAAGT
BmSpz4	
>Spz4-qPCR-F	ACTGCTCAAACGGGTGCATA
>Spz4-qPCR-R	GCAGTCATTAAGCTTGGCGG
BmSpz5	
>Spz5-qPCR-F	CAGCGTGCGTACAGAGTACA
>Spz5-qPCR-R	GCCACTGCATGTTGTGGATG
Moricin 1	
>Mor-qPCR-F	TCTTTGTTTTTATTGTGGCAATG
>Mor-qPCR-R	TTGAAAACATCGTTGGCTGT
Cecropin A	
>CecA1-qPCR-F	CTTCGTCTTCGCGTTGGT
>CecA1-qPCR-R	AAGGATTTCGCTTGCCCTAT
Defensin	
>Def-qPCR-F	GGTGCTCGTGTTTGTGTTTG
>Def-qPCR-R	CAAATCGCAGTCTCTGTTGC
Lysozyme	
>Lys-qPCR-F	GTGCATGAGCTGAGGAAACA
>Lys-qPCR-R	AGTACCGGTCGTTGATCTGG
Lebocin 3	
>Leb-qPCR-F	ACACGTACAGTGCGACAAGC
>Leb-qPCR-R	CAATGGACGCCTCGTTATTT
Gloverin 3	
>Glo3-qPCR-F	TTACGGCACCAGGGTCTTAG
>Glo3-qPCR-R	CCGGATCTCTGCTTGAAGAC
BmRPL3	
>BmRPL3-qPCR-F	CGGTGTTGTTGGATACATTGAG
>BmRPL3-qPCR-R	GCTCATCCTGCCATTTCTTACT
